# Case of Necrosis of the Lower Jaw Recovered from without Deformity

**Published:** 1844-09-07

**Authors:** William Sharp

**Affiliations:** Senior Surgeon to the Bradford Infirmary


### RECORD OF MEDICAL SCIENCE.

### CASE OF NECROSIS OF THE LOWER JAW RECOVERED FROM WITHOUT DEFORMITY.

By William Sharp, F. R. S., F. R. A. S., Senior Surgeon to the Bradford Infirmary.

The patient, a female, aged 20, was first seen by the author in the beginning of Sept. 1842, six months after the disease began, with symptoms of necrosis of the lower jaw. He extracted one of the teeth, and found a small fungous growth attached to its fang. On the 13th December, he lay the sinuses communicating between the diseased bone and the skin into one, and slightly enlarged the opening; he then drew out with the forceps the dead portion of the lower jaw, which was found to consist of about two-thirds of the entire bone, and it contained several of the alveolar processes. The author was gratified, on looking into the patient's mouth, to find that, with the exception of the tooth which he had extracted, the whole set of excellent teeth were fast, and in their proper places. The portion of bone which had been removed was exhibited to the Society.

Mr. Lloyd's experience had informed him that cases of extensive exfoliation of the jaw, where the teeth were preserved, were not at all uncommon. He had seen many such.

The President owned that he never had seen any case of the kind.

Before adjourning the Society, he informed them that Mr. Snow wished to exhibit, and was ready to explain to the members, an instrument which he had invented for the removal of fluid from the pleural cavity without the admission of air. The apparatus consisted of a trochar and canula much longer than that usually employed for paracentesis abdominis. In the middle of the canula a stop-cock is placed, which is to be turned to cut off the admission of the external air, before the trochar, which is accurately fitted to the tube, is completeiy withdrawn. The shoulder of the canula is constructed for adaptation to Reid's syringe, by which the fluid is to be removed.—*Trans. Royal Med. Chirurg. Soc. in London Med. Gaz.*

